# A Prospective Pilot Investigation of Brain Volume, White Matter Hyperintensities, and Hemorrhagic Lesions after Mild Traumatic Brain Injury

**DOI:** 10.3389/fneur.2016.00011

**Published:** 2016-02-12

**Authors:** Michael Jarrett, Roger Tam, Enedino Hernández-Torres, Nancy Martin, Warren Perera, Yinshan Zhao, Elham Shahinfard, Shiroy Dadachanji, Jack Taunton, David K. B. Li, Alexander Rauscher

**Affiliations:** ^1^UBC MRI Research Centre, University of British Columbia, Vancouver, BC, Canada; ^2^Department of Radiology, University of British Columbia, Vancouver, BC, Canada; ^3^MS/MRI Research Group, University of British Columbia, Vancouver, BC, Canada; ^4^Department of Radiology, Richmond Hospital, Richmond, BC, Canada; ^5^Department of Radiology, Burnaby Hospital, Burnaby, BC, Canada; ^6^Department of Radiology, Delta Hospital, Delta, BC, Canada; ^7^Medical Imaging Department, St Vincent’s Hospital, Melbourne, VIC, Australia; ^8^Division of Neurology, Department of Medicine, University of British Columbia, Vancouver, BC, Canada; ^9^Division of Sports Medicine, Faculty of Medicine, University of British Columbia, Vancouver, BC, Canada; ^10^Djavad Mowafaghian Centre for Brain Health, University of British Columbia, Vancouver, BC, Canada; ^11^Division of Neurology, Department of Pediatrics, University of British Columbia, Vancouver, BC, Canada

**Keywords:** concussion, mTBI, MRI, neuroimaging, brain volume, susceptibility-weighted imaging, white matter hyperintensities

## Abstract

Traumatic brain injury (TBI) is among the most common neurological disorders. Hemorrhagic lesions and white matter hyperintensities (WMH) are radiological features associated with moderate and severe TBI. Brain volume reductions have also been observed during the months following injury. In concussion, no signs of injury are observed on conventional magnetic resonance imaging (MRI), which may be a true feature of concussion or merely due to the limited sensitivity of imaging techniques used so far. Moreover, it is not known whether volume reductions are due to the resolution of trauma-related edema or a true volume loss. Forty-five collegiate-level ice hockey players (20 females) and 15 controls (9 females), 40 players underwent 3-T MRI for hemorrhages [multi-echo susceptibility-weighted imaging (SWI)], WMH (three-dimensional fluid-attenuated inversion recovery), and brain volume at the beginning and the end of the hockey season. Concussed athletes underwent additional imaging and neuropsychological testing at 3 days, 2 weeks, and 2 months after injury. At the end of the hockey season, brain volume was reduced compared to controls by 0.32% (*p* < 0.034) in the whole cohort and by 0.26% (*p* < 0.09) in the concussed athletes. Two weeks and 2 months after concussion, brain volume was reduced by −0.08% (*p* = 0.027) and −0.23% (*p* = 0.035), respectively. In athletes, the WMH were significantly closer to the interface between gray matter and white matter compared to controls. No significant changes in the number of WMH over the duration of the study were found in athletes. No microhemorrhages were detected as a result of concussion or playing a season of ice hockey. We conclude that mild TBI does not lead to transient increases in brain volume and no new microbleeds or WMH are detectable after concussion. Brain volume reductions appear by 2 weeks after concussion and persist until at least 2 months after concussion. Brain volume is reduced between the beginning and the end of the ice hockey season.

## Introduction

Traumatic brain injury (TBI) is one of the most common neurological disorders, with incidence rates >650/100,000/year (1, 2). Among the changes seen on magnetic resonance imaging (MRI) of TBI are hemorrhages, white matter hyperintensities (WMH), and brain volume reduction. These features are associated with moderate and severe brain injury (3), whereas in concussion, the most frequent type of TBI, no acute changes on conventional imaging are seen (4). Even modern MRI techniques at higher field strengths, such as gradient echo MRI or susceptibility-weighted imaging (SWI), have not detected hemorrhages in concussed patients. The absence of bleeds may be a true feature of concussion or simply a result of the limited sensitivity of imaging methods used so far. Moreover, microbleeds have a range of etiologies and are not necessarily trauma related (5, 6). Brain volume reductions have been reported to occur between the acute phases after TBI and follow-up after several months (7). However, it is not known whether these volume changes are due to the resolution of trauma-related edema (pseudoatrophy) or true volume reductions. These observations and considerations raise some questions related to conventional imaging studies of mild TBI: first, is more advanced imaging [multi-echo SWI with optimized image reconstruction (8) and 3D fluid-attenuated inversion recovery (FLAIR) at 3 T] able to detect microbleeds or WMH that are caused by concussion? And, second, does concussion lead to acute increases in brain volume and subsequent decreases that may mimic atrophy in studies that evaluate patients at two time points post injury? These questions can be answered if data from before and after the injury are available. In humans, this can only be accomplished by following a group of people at high risk of sustaining a mild TBI. Here, we worked with two ice hockey teams whom we followed over one season. In this cohort, we performed MRI pre- and postseason in all players and at 3 days, 2 weeks, and 2 months after concussion. At 3 T, we used multi-echo SWI for microbleeds, 3D FLAIR for WMH, and 3D T1-weighted scans for volumetric MRI.

## Materials and Methods

### Participants and Study Design

Twenty female and 25 male players (mean age = 21.2 ± 3.1 years) from two Canadian Interuniversity Sports ice hockey teams participated in this study. Players received baseline MRI and Sport Concussion Assessment Tool 2 (SCAT2) tests in September before the beginning of the ice hockey season (9). One physician and one non-physician, both unaffiliated with the teams, observed each preseason, regular season, and postseason game, for a total of 40 and 31 games for the men’s and women’s teams, respectively. When a suspected concussion was observed from the sideline (signs of poor balance, confusion, and disorientation), the physician removed the player from the game and performed a clinical evaluation at the rink, followed by SCAT2 in the dressing room.

Concussed players were referred to MRI and neuropsychological testing at 72 h, 2 weeks, and 2 months after concussion. Athletes were imaged after the end of the hockey season in March. Four non-concussed players completed only one time point and were excluded. Fifteen subjects (six males, nine females, age = 22.9 ± 2.3 years) from the same university were enrolled as controls for WMH and microhemorrhages and were scanned once. Inclusion criteria for controls were university students with matching age, not engaged in contact sports, and without a history of concussion or neurological condition. Five additional subjects who did not engage in contact sports were scanned four times over 6 months as healthy controls for the volume measurements. In addition, a phantom designed for quality control of the volumetric measurements in the Alzheimer’s Disease Neuroimaging Initiative (ADNI) study was scanned once a month (10). All subjects gave written informed consent prior to the study, according to the University of British Columbia research ethics board and in compliance with the Helsinki Declaration.

### Imaging

Magnetic resonance imaging data were acquired on a Philips Achieva 3T scanner equipped with an eight-channel SENSE head coil, including the following scans: (a) sagittal three-dimensional T1-weighted scan (TR = 8.1 ms, TE = 3.7 ms, flip angle = 6°, acquisition matrix = 256 × 256 × 160, field of view = 256 mm × 256 mm × 160 mm, voxel size = 1 mm × 1 mm × 1 mm, and SENSE factor of 2 along the left–right direction); (b) sagittal three-dimensional FLAIR (TR = 8000 ms, TI = 2400 ms, TE = 337 ms, flip angle = 6°, acquisition matrix = 256 × 256 × 160, field of view = 256 mm × 256 mm × 160 mm, voxel size = 1 mm × 1 mm × 1 mm, and SENSE factor of 2 along the left–right direction and 2.5 along the anterior–posterior direction); and (c) multi-echo SWI using an axial 3D gradient echo scan (TR = 36 ms, TE = 6/12/18/24/30 ms, flip angle = 17°, acquisition matrix = 440 × 222 × 64, field of view = 220 mm × 166 mm × 128 mm, acquired voxel size = 0.5 mm × 0.5 mm × 2 mm, reconstructed voxel size = 0.5 mm × 0.5 mm × 1 mm, and SENSE factor of 1.2 along the left–right direction) (8).

### Data Processing and Analysis

Susceptibility-weighted imaging data were reconstructed offline, and the SWI of the individual echoes were averaged with weights to optimize contrast between hemorrhages and surrounding tissue, assuming R2* relaxation rates of 20 ms^−1^ for white matter and 60 ms^−1^ for hemorrhage, which leads to weighting coefficients of 0.12, 0.19, 0.22, 0.24, and 0.24 (8). All other images were reconstructed by the scanner software. The FLAIR images of each concussed subject were registered to the corresponding SWI scans. The FLAIR and SWI of the non-concussed players were registered to the baseline SWI scan. All image registration was performed half-way to avoid blurring of one image more than the other. The SWI and FLAIR images were reviewed by two radiologists (Nancy Martin and Warren Perera) using custom viewing software. The radiologists (with 4 years experience), who were blinded to the control/player status, concussion status, and the chronology of the scans, reviewed the scans together. Lesions identified by consensus were digitally marked and counted. Possible lesions (questionable by one or both observers) were marked and subsequently reviewed together with the third radiologist (David K. B. Li) to confirm or exclude. To identify the distance from the WMH to the cortical gray matter and the sulcal depths, one author measured the shortest straight-line distance in 3D from the center of each WMH to the closest cortical gray matter and to the nearest sulcal depth, respectively. Lesions were also mapped into Talairach space by affine registration to the electronic Talairach atlas (11).

### Brain Volume

Two time point global brain volume changes were estimated based on the three-dimensional T1-weighted scans, using FSL’s SIENA (12–14). SIENA extracts brain and skull images from the two time point whole-head input data and measures percent brain volume change (PBVC) (15). All PBVC measurements were made against each subject’s baseline scan.

### Statistics

Statistical tests on the full season data were performed using MATLAB (2011a, The MathWorks, Inc., Natick, MA, USA). Volume changes compared to baseline were evaluated for the following groups: (1) concussed players for all post-concussion time points, (2) all concussed players postseason, (3) all non-concussed players postseason, (4) all subjects postseason with two or more WMH (observed at baseline), and (5) all subjects postseason with a maximum of one WMH at baseline. Comparison of subject and control WMH count at baseline were performed using the Wilcoxon rank-sum test. Repeated measures of the brain volume change for concussed subjects were analyzed using a mixed effect model in R (R Foundation for Statistical Computing, Vienna, Austria) (16, 17). Gender, age, SCAT2 score, and WMH count at each time point were included as fixed effects.

## Results

Over the season, 11 players were diagnosed with a concussion. Their age, gender, and number of brain lesions scores are listed in Table 1 and for the full cohort in Table 2. A total of 168 MRI sessions were performed during the study.

**Table 1:** **Demographic information and the number of WMH and hemorrhages found in the 11 concussed subjects at each time point**.

Player	Age	Gender	# of lesions at BL	# of lesions at 72 h	# of lesions at 2 weeks	# of lesions at 2 months	# of lesions at EOS
1	22	m	2	1	2	0	0
2	21	m	0	0	0	0	1
3	21	f	1	1	1	2	2
4	19	f	0	X	0	0	X
5	22	f	1	1	1	1	0
6	21	f	0	0	0	0	0
7	22	m	1	1	1	0	0
8	24	m	6 (+1 bleed)	X	6 (+1 bleed)	X	X
9	19	f	4	X	4	4	4
10	19	f	0	0	0	0	0
11	23	m	5	6	6	X	X

*All lesions were WMH, with the exception of one hemorrhage in subject 8, which was already present at the preseason scan. X indicates a missed follow-up*.

**Table 2:** **Demographic details and lesion counts for all players who underwent both pre- and postseason MRI scans**.

Subject	Concussed (Y/N)	Sex	Age	Preseason MWHI	Postseason MWHI
1	Y	M	22	2	0
2	Y	M	21	0	1
3	Y	F	21	1	2
4	Y	F	19	0	X
5	Y	F	22	1	0
6	Y	F	21	0	0
7	Y	M	22	1	0
8	Y	M	24	7	X
9	Y	F	19	4	4
10	Y	F	19	0	0
11	Y	M	23	5	X
12	N	F	23	3	3
13	N	M	21	0	0
14	N	F	18	5	5
15	N	F	20	10	11
16	N	F	18	0	0
17	N	F	18	5	3
18	N	M	21	3	3
19	N	M	24	2	3
20	N	F	21	1	1
21	N	M	22	2	5
22	N	M	25	2	2
23	N	M	22	8	9
24	N	M	21	1	1
25	N	F	19	0	1
26	N	F	18	1	1
27	N	M	22	32	29
28	N	M	20	14	13
29	N	M	21	0	0
30	N	M	21	3	4
31	N	F	20	2	2
32	N	M	22	6	5
33	N	F	18	3	0
34	N	M	23	1	1
35	N	F	17	0	0
36	N	M	20	1	1
37	N	F	19	3	2
38	N	F	17	0	1
39	N	M	21	2	2
40	N	M	23	6	6
41	N	F	36	6	7

### Lesions

**Figure 1:**
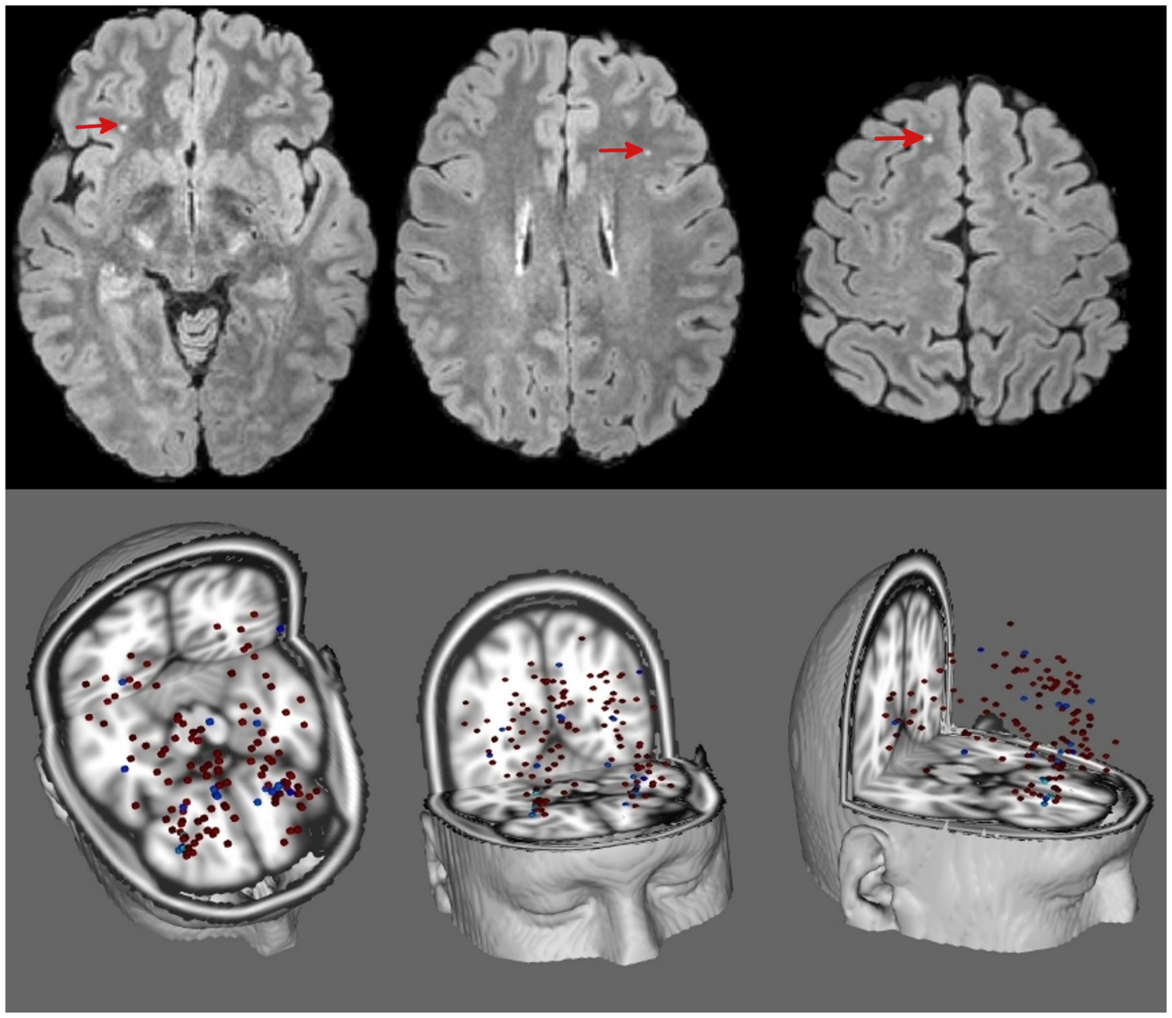
**Top**: T2-weighted FLAIR of a 20-year-old non-concussed male ice hockey player showing punctuate WMH (red arrow heads). **Bottom**: composite image of all WMH of players (red, from 41 players) and controls (blue, from 15 controls) registered to a standard brain.

**Figure 2:**
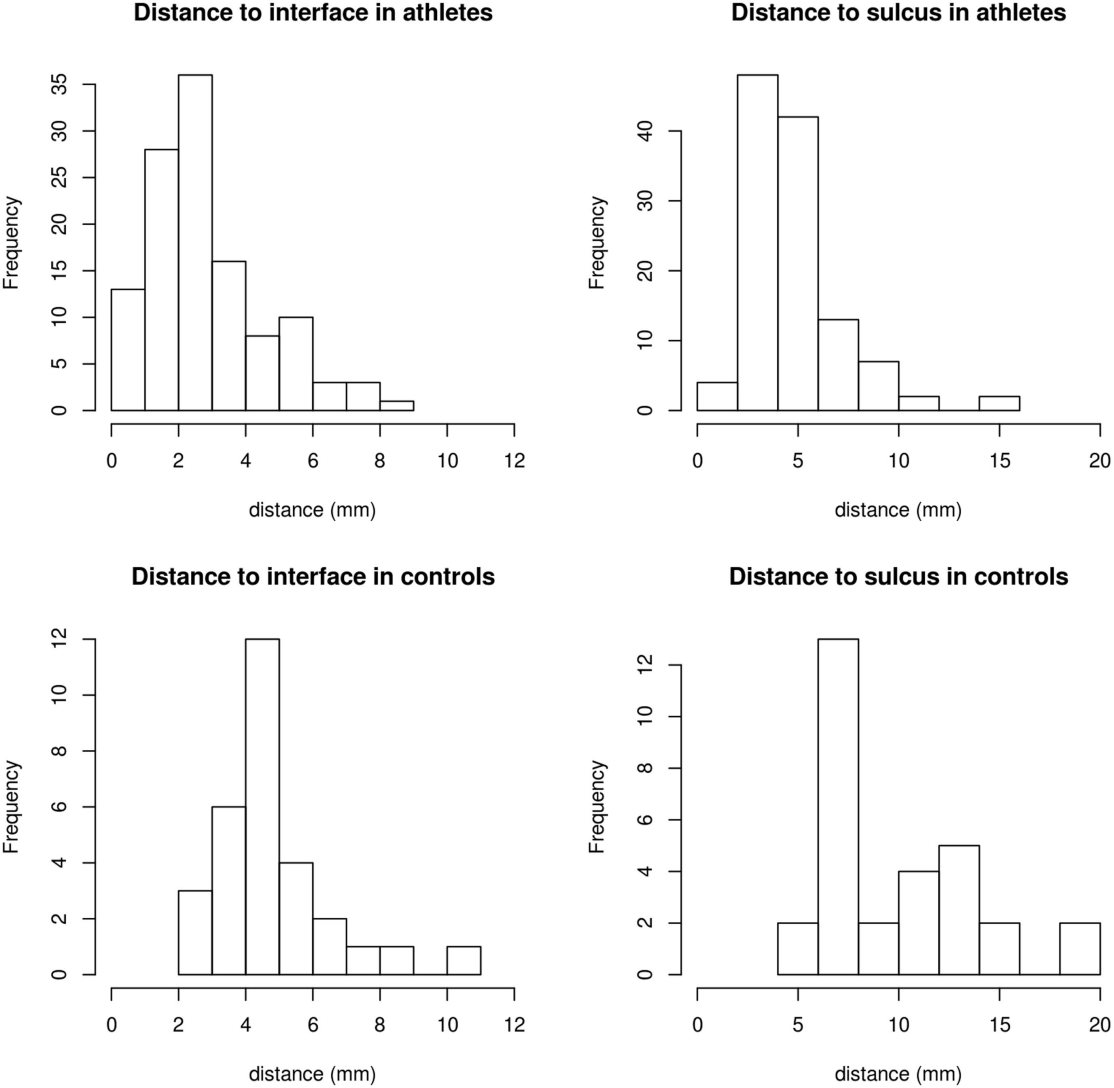
**Distance from WMH to the cortical interface and the nearest depth of sulcus in athletes and controls, respectively**. In athletes, mean, median first quartile, and third quartile were 2.6, 2.5, 1.5, and 3.9 mm for the distance to the nearest GM/WM interface and 4.4, 4.2, 3.1, and 5.6 mm to the nearest sulcal depth. In controls, the distances were 5.2, 5.0, 4.0, and 5.7 mm to the nearest WM/GM interface and 10.0, 8.5, 7.0, and 12.5 mm to the sulcal depths.

An example of punctuate WMH in non-concussed ice hockey player is shown in Figure 1. The number of WMH and hemorrhages of each concussed subject at each available time point is listed in Table 1. The median number of WMH for all hockey players was 3.5 ± 5.5 (median 2, max = 32, and min = 0). The number of WMH exhibited some variability over time but no significant association with concussion. The non-concussed players similarly showed no difference in WMH between pre- and postseason scans. The distance (mean ± SD) of the WMH to the cortical gray matter and the sulcal depths was 2.6 ± 2.6 and 4.3 ± 3.4 mm, respectively. WMH were principally found in the front lobe (75.2%). The only hemorrhage detected in the whole cohort had already been present at the preseason baseline scan (subject 8). Since there were no significant changes in lesion number over the ice hockey season, comparisons with lesion number and location in controls were performed for the baseline data. In the control cohort, we found on average 2.1 ± 2.2 (median = 2, max = 6, and min = 0) WMH and no microhemorrhages. In the controls, the distance between WMH and the closest cortical GM and the sulcal depths was 5.2 ± 1.7 and 10.7 ± 3.8 mm, respectively, which is significantly (*p* < 0.002) (with correction for multiple comparison for the two measurements of distances to the GM/WM interface and the sulcal depth) larger than in the athletes (with correction for multiple comparison for the two measurements of distances to the GM/WM interface and the sulcal depth), and the majority of WMH (56%) were located in the frontal lobe, which accounts for approximately 33% of total brain volume (Figure 2).

### Volume

**Figure 3:**
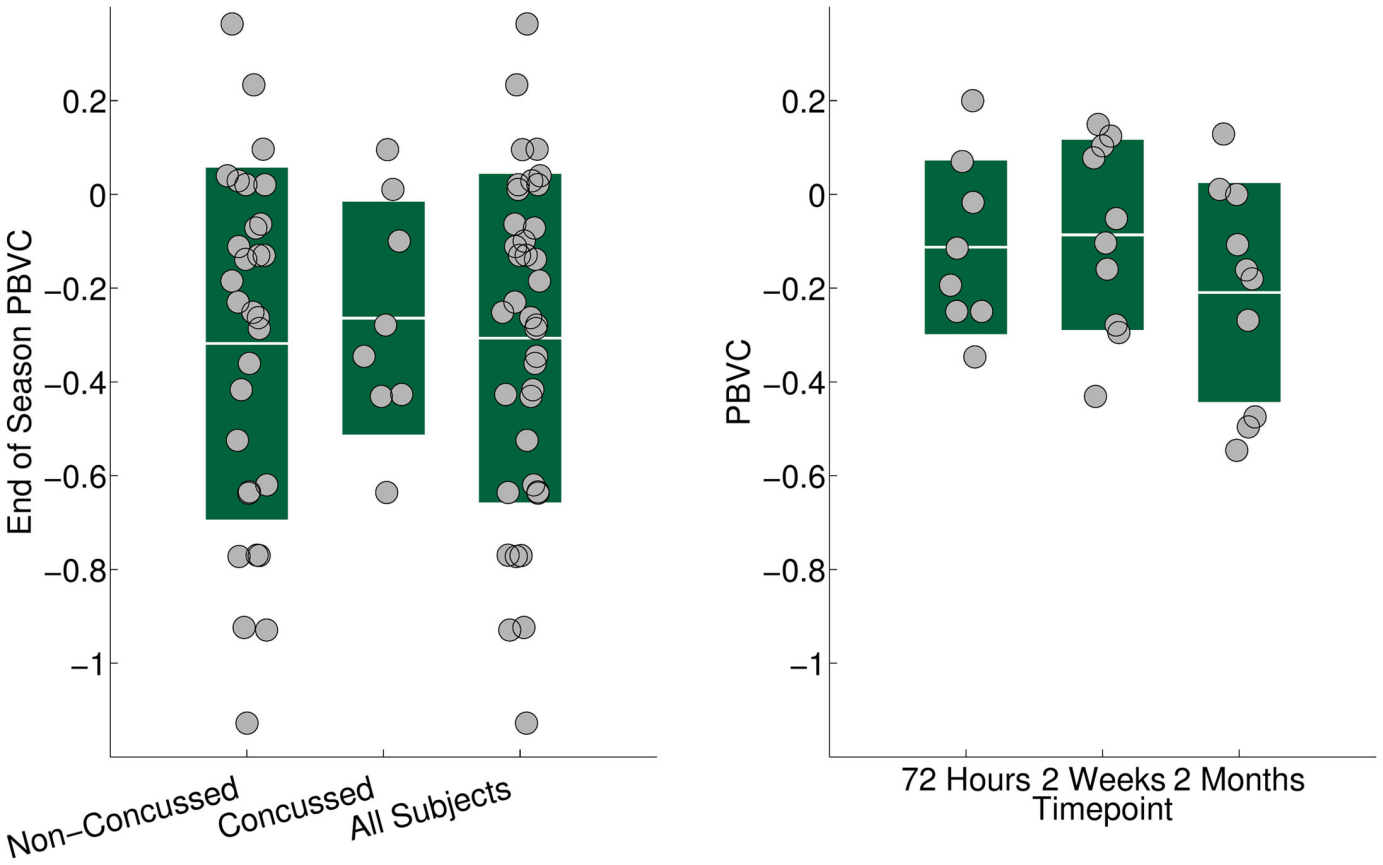
**Left**: brain volume changes in percent at the end of the hockey season relative to the preseason volume in the non-concussed, the concussed, and the whole cohort. Solid bars show the SD. All three groups (concussed, non-concussed, and whole cohort) showed a significant (*p* < 0.01) reduction in brain volume. The volume reduction in the whole cohort was not driven by the concussed players. **Right**: reduction in brain volume in 11 concussed ice hockey players 72 h, 2 weeks, and 2 months after concussion, compared to preseason baseline. At 2 months, the brain volume was reduced significantly (*p* = 0.016) by 0.23%.

In the control subjects, PBVC changes between baseline and subsequent time points were positive but not significant [+0.07% (*p* = 0.36), +0.18% (*p* = 0.11), and +0.07% (*p* = 0.45)]. Compared to controls, PBVC of the concussed players were not significantly reduced 3 days after concussion (−0.11%, *p* = 0.15). Two weeks and 2 months after concussion, PBVC of −0.08% (*p* = 0.027) and −0.23% (*p* = 0.035) were significant compared to controls. In the non-concussed players, the full season PBVC of −0.32% was significantly more than in the final control time point (*p* = 0.034). There was no significant difference in PBVC between concussed and non-concussed athletes at the end of the season (Figure 3). No significant differences in PBVC were found between male and female players (*p* = 0.77). The ADNI phantom scans showed no scanner-related changes in measured phantom geometry.

Based on the mixed effect model, the only factor found significantly related to PBVC of concussed subjects was time after injury (*p* = 0.009). Age (*p* = 0.09), gender (*p* = 0.4), SCAT2 (*p* = 0.3), and WMH count (*p* = 0.07) were not found to have a significant effect.

## Discussion

The main findings of this prospective neuroimaging study on mild TBI were (1) that no volume changes were observed at 3 days and 2 weeks post injury, but both concussed and non-concussed athletes exhibited reduction in brain volume over the course of one season; (2) that neither concussion nor playing a season of ice hockey led to detectable microbleeds; and (3) that in athletes, WMH were more numerous and significantly closer to the GM–WM interface compared to controls.

### Volume

The volume changes in both the concussed and non-concussed players were small (corresponding to 3 cm^3^ if a typical brain volume is assumed), yet significant. This reduction is in line with previous studies in mild TBI, where reductions in volume by about 7.6 cm^3^ within 1 year were measured (7, 18). The discrepancy between volumetric MRI and SCAT2 suggests that there may be subtle long-lasting effects that are not detected by SCAT2. Since this pilot study was conducted, SCAT3 has been developed and recommended for sport concussion (19). However, both SCAT2 and SCAT3 were designed as simple tools for the identification of concussions rather than as neuropsychological tests. Furthermore, while certain aspects of concussion may be better captured with subcategories of SCAT2 or the newer SCAT3, the statistical power of this neuroimaging study is too low to compare imaging outcomes with several subscores. Future studies should compare neuroimaging with more sensitive neuropsychological tests for serial follow-up designed for postconcussive symptoms. The pre- and postseason scans for this study were performed for all subjects during two brief windows several months apart. With such design, variability in scanner performance may be responsible for changes in measured brain volume. In addition to the five healthy controls, who showed no volume change, the MRI scanner performance at our institution is assessed by regular scans of a phantom for the ADNI study (10). These ADNI phantom scans showed no signs of scanner drift that may lead to errors in volume measurements. The data acquired 3 days after concussion were collected when dehydration from the physical activity may be present. We cannot ignore the possibility of a systematic short-term physiological state being responsible for the observed volume change, such as differences in hydration levels at the different measured time points. One study has found that a lack of fluid intake prior to MRI can result in a decrease in brain volume by 0.55% (20). On the other hand, two recent studies have investigated whole brain volume change in dehydrated athletes using both SIENA and a manual method and found no measurable change (21, 22), while a third study showed a decrease of brain volume by 0.36% (23). Using accelerometers (24) to relate volume changes to the impact doses sustained during a season of contact sports would provide more insight into the relationship, if any, between subclinical impacts and brain volume changes. It is an important finding of the present study that there were no increases in brain volume at 72 h and 2 weeks after concussion. Such changes could occur from diffuse edema and swelling associated with injury and be a potential confounding factor in brain volume studies where subjects are scanned short after injury and again several months later, but not prior to the injury. In such studies, resolution of edema may mimic atrophy (pseudoatrophy). We must also note that the small volume changes observed here do not necessarily correspond to neuropsychiatric deficits.

### Microhemorrhages and WMH

Microhemorrhages have not been reported in concussion; this may be a feature of concussion or simply a result of the limited sensitivity of imaging methods used in concussion so far. The new multi-echo SWI technique used for this study has 40% better signal-to-noise ratio compared to its single echo counterpart (8). Moreover, data were acquired at 3 T and offline processing of the data was optimized for the visualization of hemorrhages. The absence of concussion-related bleeds even on such an advanced scan supports the notion that hemorrhages are not a predominant feature of concussion.

However, the lack of hemorrhagic injury in this cohort does not mean that contact sports, such as ice hockey, are harmless. Neither microhemorrhages nor WMH are unequivocal signs of recent brain injury. Microbleeds and WMH are associated with various conditions, including TBI, and they may persist for several years (6, 25). Interestingly, using a 3D FLAIR scan with 1 mm^3^ isotropic resolution at 3 T, we found more WMH than reported previously using 2D approaches. For example, only one WMH was found in a group of 65 healthy subjects between 16 and 25 years, using 2D FLAIR at 1.5 T (26). It should be noted that the visual detection of punctuate hyperintensities is inherently subjective, which may account for some of the variability in WMH counts across time points (27). While the number of WMH was elevated but not significantly higher in the athletes compared to the controls, their location was significantly closer to the gray matter and the sulcal depths. This proximity to the GM/WM interface suggests that the WMH in athletes are more likely impact related (28, 29). Since there were no significant changes in WMH over time, the comparison between athletes and controls was performed for the baseline data only.

It was suggested that chronic changes in the brains of some athletes may be a result from multiple hits. A recent postmortem study on the brains of 85 people with histories of repetitive mild TBI, for instance, found that in the subgroup of 35 professional American football players, pathological signs of chronic traumatic encephalopathy (CTE) were present in 34 players (30). CTE is thought to be a progressive neurodegeneration characterized by tau neurofibrillary tangles which, starting from the depths of the cerebral sulci, spread over larger cortical areas as the disease progresses (31–33). In these 34 football players, the stage of CTE correlated with the number of years played but not with the number of concussions. In the present study, the majority of the WMH in the athletes were found within 5 mm of the nearest sulcal depth and within 3 mm of the white matter/gray matter interface; the WMH were not only more frequent but also significantly closer to the GM/WM interface than in the controls. Recent studies also demonstrated changes in white matter integrity in non-concussed athletes over one season of impact sports using diffusion tensor imaging (34, 35). These studies support the hypothesis that an accumulation of subconcussive hits can have an appreciable effect on brain structure. It should be emphasized that the postmortem studies on CTE have a heavy selection bias, as usually only athletes with clinical symptoms donate their brains for this type of research.

### Limitations

This study had some limitations. The variability in the counts of WMH seen pre- and postseason, as well as post-concussion may have limited the ability to detect change over time and following injury. Partial volume averaging was minimized by the 3D acquisition with 1 mm isotropic resolution and two radiologists worked by consensus in order to minimize inter- and intra-rater variability. The variability in WMH counts highlights the subjective nature and the difficulty in identifying small punctuate lesions, particularly when the observers were blinded to the chronology of the scans. The high sensitivity of the multi-echo SWI and the 3D FLAIR comes at the expense of limited comparability with studies that use standard clinical MRI protocols, although SWI and 3D FLAIR are now commonly available sequences. The sample size of concussed athletes is relatively small, which precludes an in-depth analysis of the relationship between neuropsychological scores and the number and location of WMH or any differences between male and female players. This also may have been a factor in the lack of change in SCAT2 scores. In contrast, these significant changes seen in a small cohort encourage future prospective studies on mild TBI in high-risk groups. The prospective nature of the study limited the sample size but allowed us to directly compare players before and after playing a season of hockey and before and after injury, greatly reducing the influence of intersubject variability encountered in cross-sectional studies. Finally, not all subjects had all MRI scans or all neuropsychological tests. However, the statistical power is similar at all time points with eight out of 11 concussed subjects scanned at 72 h, all 11 scanned at 2 weeks, and nine out of 11 scanned at 2 months. The time points after concussion were chosen to sample the acute phase after concussion, the phase after which people are normally regarded as recovered (2 weeks), and a follow-up several weeks after full recovery. The 72-h time point was the earliest feasible time point to perform post-injury MRI. In particular, in the early phase after injury, a different choice of time points may have an influence on the results. Our choice of time points after concussion is not unusual, however (36). While a prospective study in a cohort with a high risk of sustaining a concussion is much closer to a real life scenario than any animal work, biomechanical aspects specific to ice hockey may limit the generalizability of our findings. Additional studies in other high-risk cohorts, such as American football, will shed more light on the issue of generalizability.

## Conclusion

The present study demonstrates that concussions do not cause acute increases in brain volume and that even with advanced MRI at high field strength, no microhemorrhages are detected. The findings also suggest that playing ice hockey may lead to observable volume changes in the athletes’ brains, irrespective of their concussion status, indicating that better monitoring of players and more protective measures may be warranted. However, in this study, the degree of change was small and factors other than repetitive impact from playing ice hockey cannot be ruled out. Future prospective studies should address whether these changes accumulate over consecutive seasons of contact sports and whether they are reversible once athletes stop playing competitively.

## Author Contributions

AR, JT, and DL designed the study. AR and DL designed the imaging protocol. SD coordinated the study and performed neuropsychological testing. MJ and YZ performed statistical analysis. RT designed the lesion marking workflow, software, and performed quality control and visual inspection of all marked lesions. MJ, EH-T, RT, and ES performed image reconstruction and preprocessing. NM and WP marked lesions. AR and DL supervised imaging and image analysis. AR and MJ conducted literature research. MJ and AR wrote the first draft. All authors contributed to the writing of the paper. All authors read and approved the final manuscript.

## Conflict of Interest Statement

The authors declare that the research was conducted in the absence of any commercial or financial relationships that could be construed as a potential conflict of interest.
